# The Role of Gram-Positive Surface Proteins in Bacterial Niche- and Host-Specialization

**DOI:** 10.3389/fmicb.2020.594737

**Published:** 2020-10-29

**Authors:** Amy C. Pickering, J. Ross Fitzgerald

**Affiliations:** The Roslin Institute and Edinburgh Infectious Diseases, University of Edinburgh, Easter Bush Campus, Edinburgh, United Kingdom

**Keywords:** specialized, niche, host, surface protein, adaptation, bacteria, interaction

## Abstract

Gram-positive bacterial pathogens have an array of proteins on their cell surface that mediate interactions with the host environment. In particular, bacterial cell wall-associated (CWA) proteins play key roles in both colonization and pathogenesis. Furthermore, some CWA proteins promote specialization for host-species or mediate colonization of specific anatomical niches within a host. In this mini review, we provide examples of the many ways by which major pathogens, such as Staphylococci, Streptococci and *Listeria monocytogenes*, utilize CWA proteins for both host- and niche-specialization. We describe different biological mechanisms mediated by CWA proteins including: the acquisition of iron from hemoglobin in the bloodstream, adherence to and invasion of host cells, and innate immune evasion through binding to the plasma proteins fibrinogen, immunoglobulin G, and complement. We also discuss the limitations of using animal models for understanding the role of specific CWA proteins in host-specialization and how transformative technologies, such as CRISPR-Cas, offer tremendous potential for developing transgenic models that simulate the host environment of interest. Improved understanding of the role of CWA proteins in niche- or host-specificity will allow the design of new therapeutic approaches which target key host–pathogen interactions underpinning Gram-positive bacterial infections.

## Introduction

Bacteria have typically evolved to occupy particular niches within host-species or the environment. For example, *Streptococcus uberis* and some clones of *Staphylococcus aureus* are specialized for infection of the bovine mammary gland and *Listeria monocytogenes* has a tropism for transcytosis of the placenta and intestinal epithelia (Lecuit et al., 2004). Furthermore, bacterial pathogens have adapted to colonize either a single host-species (specialist) or multiple host-species (generalist) (Woolhouse et al., 2001; Bäumler and Fang, 2013). For example, the human pathogen *Streptococcus pyogenes*, associated with infections such as pharyngitis, skin infections, rheumatic fever and necrotizing fasciitis, and the equine pathogen *Streptococcus equi* subsp. *equi*, associated with the respiratory disease strangles, have a very restricted host-specialization. In contrast, the multi-host “generalist” *S. aureus* is associated with an array of different infections in humans, livestock and wild animal species (Sheppard et al., 2018). However, individual clonal lineages or subtypes of *S. aureus* have evolved the capacity to infect distinct host-species following a host-switch event (Lowder et al., 2009; Fitzgerald, 2012; Richardson et al., 2018; Haag et al., 2019). Adaptation to a novel niche or host-species involves multiple evolutionary mechanisms including mutation, recombination and horizontal gene transfer. Comparative genomic analysis of *S. aureus* from different host-species has identified genetic signatures affecting cell surface proteins, marked by gene acquisition, diversification and loss of function events, suggesting a key role for cell surface proteins in host- and niche- specialization (Herron-Olson et al., 2007; Ben Zakour et al., 2008; Lowder et al., 2009; Guinane et al., 2010; Resch et al., 2013; Spoor et al., 2015). Signatures of adaptation affecting the cell envelope may reveal pathways that could be disrupted therapeutically. For instance, *S. aureus* has undergone several host-switch events from humans into rabbits underpinned by natural adaptive mutations in a gene encoding DltB, a membrane-associated protein involved in D-Ala modification of wall teichoic acids (Viana et al., 2015). These data highlighted the key importance of DltB in host–pathogen interactions, a discovery reinforced by a subsequent study that identified DltB as a novel druggable target (Pasquina et al., 2016).

**FIGURE 1:**
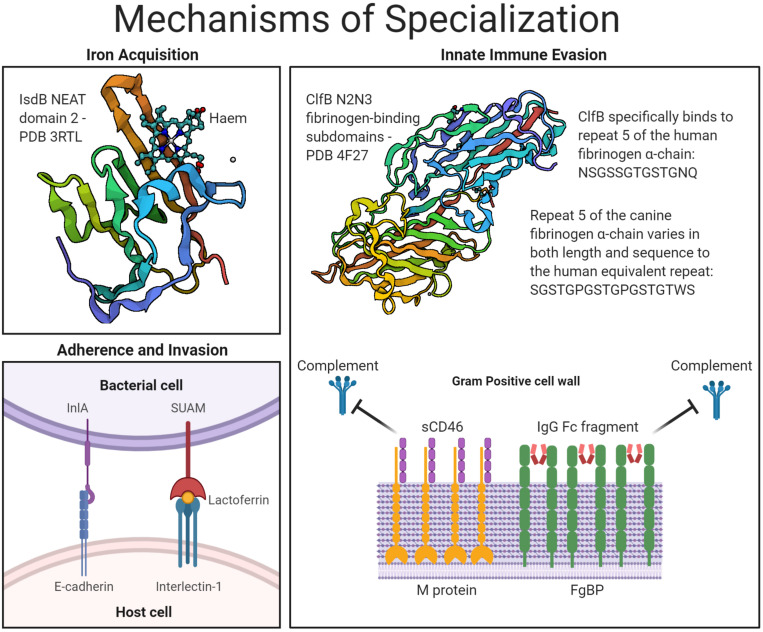
Mechanisms of host- and niche-specialization by Gram-positive surface proteins. An overview of the CWA bacterial proteins described under the three key headings of: iron acquisition, adherence and invasion, and innate immune evasion. Protein structures accessed from PDB files 3RTL, for IsdB in complex with haem (Gaudin et al., 2011), and 4F27, for ClfB in complex with fibrinogen (Xiang et al., 2012).

The aim of this mini review is to highlight the role of cell wall-associated (CWA) proteins of Gram-positive bacteria in overcoming host- and niche-specific barriers to infection. We provide examples of CWA proteins that are either covalently bound to cell wall peptidoglycan, or indirectly attached to the cell wall via non-covalent interactions with wall teichoic acids. Important host- or niche-specific CWA protein interactions underpinning biological mechanisms are described including: acquisition of iron from hemoglobin, adherence to and invasion of host cells, and evasion of the innate immune response (Figure 1). Understanding the mechanisms by which bacteria adapt to different niches or host-species can reveal critical host–pathogen interactions that could potentially be targeted to develop novel therapeutic approaches. However, demonstration of the *in vivo* importance of a host-specific CWA protein interaction is often challenging outside of the natural host. Here, we also discuss the potential for the design of transgenic models that express host-specialized surface protein receptors and would facilitate studies into the role of host- or niche-specific interactions in bacterial pathogenesis.

## Nutrient Acquisition of Iron From Hemoglobin

Bacterial adaptation to a particular host-species or niche often requires modification of surface proteins to allow them to interact with distinct ligands or polymorphic host receptors. Iron is an essential nutrient required as a co-factor for amino acid biosynthesis, the TCA cycle, DNA replication, cellular respiration, and electron transport (Sheldon and Heinrichs, 2015). In the host, iron is often sequestered in molecules such as hemoglobin in the blood and lactoferrin in mucosal secretions and milk. During bacterial infection, iron availability is actively decreased by limiting iron uptake from food digestion in a process termed nutritional immunity (Weinberg, 1974). However, bacteria have evolved numerous mechanisms, some of which involve utilization of surface proteins, to overcome this limitation and to successfully compete with the host for iron.

### Heme Acquisition – Iron-Regulated Surface-Determinant Pathway

One such mechanism involves the iron-regulated surface-determinant (Isd) pathway present in a number of Gram-positive bacteria, including *S. aureus*, *S. pyogenes*, *Bacillus anthracis*, and *L. monocytogenes* (Sheldon and Heinrichs, 2015). In *S. aureus*, the Isd pathway is involved in acquisition of iron from hemoglobin and myoglobin in the bloodstream, allowing *S. aureus* to rapidly adapt to low iron conditions (Torres et al., 2006). *S. aureus* also responds to low iron conditions by altering the expression of metabolic proteins to generate a more acidic environment, promoting the release of iron from host proteins (Friedman et al., 2006). The Isd pathway is represented by multiple cell envelope-associated proteins encoded in an operon, with the near iron transporter (NEAT) domain 1 of the CWA protein IsdB interacting directly with hemoglobin (Torres et al., 2006). Heme is then transferred to NEAT domain 2 of IsdB and along a chain of Isd proteins concluding in heme binding to the IsdE membrane-associated lipoprotein. This allows IsdF-mediated translocation of the heme iron into the bacterial cytoplasm for use in metabolism (Muryoi et al., 2008; Zhu et al., 2008; Bowden et al., 2014; Gianquinto et al., 2019). Notably, IsdB is a host-specialized CWA protein with enhanced binding affinity for human hemoglobin, in comparison to hemoglobin from other mammalian sources (Pishchany et al., 2010). Strikingly, mice expressing a human hemoglobin protein have increased susceptibility to *S. aureus* infection in comparison to those expressing the native murine hemoglobin (Pishchany et al., 2010). The crystal structure of IsdB, in complex with human hemoglobin, revealed that binding results in conformational changes that displace heme from the β-subunits and subsequently the α-subunits of hemoglobin (Bowden et al., 2018). This IsdB-hemoglobin interface exhibits signatures of positive selection during the evolution of primates, involving both the α- and β-globins, suggesting that the role of IsdB in human-specialization is part of a long evolutionary relationship between *S. aureus* and hemoglobin (Choby et al., 2018). The specialization of IsdB to human hemoglobin, is similarly observed in the closely related *Staphylococcus argenteus* and *Staphylococcus schweitzeri* species, in addition to the more distantly related human-specialized *Staphylococcus lugdunensis* (Zapotoczna et al., 2012; Choby et al., 2018). Recently, a novel iron acquisition system in *S. lugdunensis*, dependent on an ECF-type transporter, has also demonstrated host-restriction based on the inability to lyse non-human erythrocytes to release hemoproteins (Jochim et al., 2020). These findings highlight how the co-evolution of bacterium and host can lead to bacterial adaptation to a key nutrient source in a particular host.

## Adherence and Invasion of Host Cells

In addition to nutrient acquisition, all bacterial pathogens must overcome an array of anatomical, physiological and immunological barriers to colonize and establish infection. Adherence to and invasion of host cells is an important strategy for bacterial colonization, immune evasion, and dissemination. Gram-positive bacteria exploit a range of mechanisms to mediate binding and uptake by different cell types (Ribet and Cossart, 2015; Josse et al., 2017). Such interactions may promote establishment in a particular niche found across multiple host-species or may require diversification, to facilitate binding to tissue or cells that have polymorphic ligands or receptors in a distinct host-species.

### Internalin A of *Listeria monocytogenes*

A well characterized example of a CWA protein mediating both niche- and host-specialized cellular internalization is the internalin A (InlA) protein of *L. monocytogenes*, a veterinary pathogen that causes encephalitis in ruminants and a highly virulent foodborne pathogen that can cause miscarriage, stillbirth, or premature labor in pregnant women or meningitis in newborns (Williams et al., 2011; Roulo et al., 2014). InlA interacts with the N-terminal region of E-cadherin expressed by epithelial cells, including those present on the placental villous trophoblast barrier, promoting niche-specialization (Mengaud et al., 1996; Schubert et al., 2002; Lecuit et al., 2004). Primarily, this interaction allows *L. monocytogenes*, where E-cadherin is exposed, to pass through the intestinal epithelial barrier via transcytosis either on the luminal surface around intestinal goblet cells or intercellular junctions that form as part of normal gut homeostasis (Pentecost et al., 2006; Nikitas et al., 2011). It has been demonstrated that heterologous expression of InlA confers the capacity for internalization to the invasion-deficient *Listeria innocua*, and that natural premature stop codons in the *inlA* gene of *L. monocytogenes* clinical isolates are associated with a reduced capacity to invade intestinal epithelium and an increased infective dose in experimental models (Gaillard et al., 1991; Van Stelten et al., 2011; Su et al., 2019).

Determination of the crystal structure of the InlA interaction with E-cadherin provided evidence for host-specialization with stronger binding observed to human compared to murine E-cadherin (Lecuit et al., 1999; Schubert et al., 2002). Elegant mutational experiments demonstrated that a single proline residue at position 16 of E-cadherin was sufficient to alter host-tropism (Lecuit et al., 1999). Presence of Pro16 in E-cadherin, as naturally occurs in humans, rabbits, and guinea pigs, facilitates strong binding, whereas Glu16, as occurs in rodents, leads to a reduced affinity (Lecuit et al., 1999). Of note, rabbits are considered to be the natural hosts of *L. monocytogenes* with cows also encoding Pro16 in bovine E-cadherin (Murray et al., 1926; Zimin et al., 2009). InlA of *L. monocytogenes* has therefore evolved to promote both niche- and host-specialized invasion of intestinal and placental epithelial cells in the natural rabbit host, and conserved residues in human and bovine E-cadherin underpin the capacity for zoonotic infection.

### *Streptococcus uberis* Adhesion Molecule

*Streptococcus uberis*, a leading cause of bovine mastitis, has also evolved a niche- and host-specialized mechanism of adherence and invasion. Lactoferrin is an iron-sequestering glycoprotein with antimicrobial properties that is highly expressed in milk during bovine mastitis (Harmon et al., 1976; Hagiwara et al., 2003; Chaneton et al., 2008). Due to its abundance and importance in host defense, many bacterial pathogens have evolved mechanisms for interacting with lactoferrin (Valenti and Antonini, 2005; Lu et al., 2020). *S. uberis* encodes two lactoferrin-binding proteins that demonstrate preference for bovine lactoferrin, in comparison to the human variant, and promote evasion of the antimicrobial properties of lactoferrin in the mammary gland niche (Fang and Oliver, 1999; Moshynskyy et al., 2003). In particular, *S. uberis* adhesion molecule (SUAM) uses lactoferrin as a cross-bridge with host interlectin-1 to promote adherence to and internalization of *S. uberis* into bovine mammary epithelial cells (Fang et al., 2000; Moshynskyy et al., 2003; Almeida et al., 2006; Patel et al., 2009; Chen et al., 2011). A mutant *S. uberis* strain deficient in SUAM expression was attenuated for virulence in an experimentally infected mammary gland, suggesting a key role for SUAM in the pathogenesis and niche-specificity of *S. uberis* (Patel et al., 2009; Almeida et al., 2015).

## Innate Immune Evasion

In addition to the acquisition of key nutrients and the subversion of host cells, overcoming the host immune response is critical for the pathogenesis of Gram-positive bacteria. A wide array of innate immune mechanisms have evolved to prevent bacterial infections including the development of blood clots at wound sites and damaged blood vessels, and the activation of the complement cascade leading to intracellular killing by phagocytic cells such as neutrophils, macrophages, and dendritic cells. In response, Gram-positive bacteria can utilize surface proteins to disrupt these immune mechanisms in a niche or host-specialized manner by interacting with host proteins such as the plasma glycoprotein fibrinogen, to inhibit both hemostasis and phagocytosis, and complement factors such as factor H and CD46, to inhibit complement deposition.

### Staphylococcal Interactions With Fibrinogen

Fibrinogen plays a central role in hemostasis and contains α- β- and γ-chains as a dimer of trimers (Mosesson, 2005). The ubiquity and abundance of fibrinogen has led a wide range of bacterial pathogens to develop ways of subverting fibrinogen for adherence to host cells, abscess formation, and immune evasion (Ko and Flick, 2016; Thomer et al., 2016). Staphylococcal species, including *S. aureus*, *S. lugdunensis*, and *S. pseudintermedius*, commonly adhere to the C-terminal of the fibrinogen γ-chain to provide a generalized but host-specific interaction, which interferes with fibrinogen-mediated coagulation and platelet aggregation (McDevitt et al., 1997; Geoghegan et al., 2010; Pietrocola et al., 2013). In the case of clumping factor A (ClfA) of *S. aureus*, the fibrinogen γ-chain binding is equivalent for human, canine, feline, murine, and porcine fibrinogen but demonstrates reduced binding to bovine fibrinogen and no detectable ovine fibrinogen-binding, due to a single amino acid substitution in the ovine fibrinogen γ-chain (Geoghegan et al., 2010). This host-specific fibrinogen-interaction of ClfA is essential for *S. aureus*-mediated sepsis, in murine infection models, as well as mediating phagocytosis inhibition and bacterial aggregation, key innate immune evasion strategies of *S. aureus* (Higgins et al., 2006; Flick et al., 2013; Claes et al., 2018).

Additional Staphylococcal CWA proteins demonstrate a more host-specialized fibrinogen interaction with the repeat region of the fibrinogen α-chain, which exhibits both inter- and intraspecies variation (Murakawa et al., 1993; Inaba et al., 2008; Pickering et al., 2019). For example, clumping factor B (ClfB) of *S. aureus* interacts solely with repeat 5 of the human fibrinogen α-chain, promoting human-specialized platelet aggregation, with *S. pseudintermedius* surface protein L (SpsL) exhibiting canine-specialized high-affinity binding to the repeat region of the canine fibrinogen α-chain (Miajlovic et al., 2007; Walsh et al., 2008; Pickering et al., 2019). SpsL is unique in exhibiting both canine-specific binding to the repeat region of the fibrinogen α-chain and a secondary weaker binding interaction that is also observed with human fibrinogen (Bannoehr et al., 2011; Pickering et al., 2019). This canine-specialized fibrinogen interaction promotes *S. pseudintermedius* aggregation and phagocytosis inhibition by neutrophils, demonstrating the role of fibrinogen-binding as a host-specialized immune evasion strategy of *S. pseudintermedius* (Pickering et al., 2019). Adherence to the fibrinogen α-chain is also utilized by the Srr1 and Srr2 glycoproteins of *Streptococcus agalactiae* for host-specialization, with specific binding to human repeats 6-8, demonstrating that this region of fibrinogen is the target of host-specialization for an array of Gram-positive bacteria (Seo et al., 2013).

### M protein of *Streptococcus pyogenes*

*Streptococcus pyogenes* is a major human-specific pathogen that utilizes a range of innate immune evasion strategies to cause acute pharyngitis and impetigo as well as severe invasive infections such as necrotizing fasciitis (Laabei and Ermert, 2019). The most abundant CWA protein presented on the surface of *S. pyogenes* is the M protein, with certain M protein serotypes associated with specific niches such as the throat or skin (Fischetti, 1989; Cunningham, 2000). The M protein has an array of host ligands that promote both host cell adherence and immune evasion (Fischetti, 1989). The binding of M protein to complement components such as factor H, C4BP and CD46 all lead to complement inhibition (Horstmann et al., 1988; Thern et al., 1995). In the case of CD46 complement regulatory protein, which aids the cleavage of complement factors C3b and C4b on host cells, M protein has been demonstrated to interact in a human-specific manner (Riley-Vargas et al., 2004; Lövkvist et al., 2008). The interaction of M protein with human cellular CD46 mediates binding to keratinocytes and invasion of lung epithelial cells ultimately leading to cell death (Okada et al., 1995; Rezcallah et al., 2005). During epithelial cell apoptosis, soluble CD46 is shed from the host cell leading to reduced killing of *S. pyogenes* in whole blood due to its interaction with M protein (Lövkvist et al., 2008). This immune evasion mechanism is human-specialized as evidenced by increased whole blood survival in transgenic mice expressing human CD46 (Lövkvist et al., 2008). These human-expressing CD46 mice were also more susceptible to experimental infection with increased mortality and bacterial levels during a bloodstream infection and the development of necrotizing fasciitis after subcutaneous injection (Lövkvist et al., 2008; Matsui et al., 2009). *S. pyogenes* has therefore developed a human-specialized immune evasion strategy associated with complement inhibition.

### Immunoglobulin Binding by *Streptococcus equi* subsp. *equi*

A final example of host-specific immune evasion is the fibrinogen-binding protein (FgBP) of the equine-specific *S. equi* subsp. *equi*, the cause of the respiratory disease strangles in horses. FgBP is a CWA protein which was originally characterized as a fibrinogen-binding protein with preference for equine Fg and as a protective antigen in a murine infection model (Meehan et al., 1998, 2000). However, it was later identified as an IgG-binding protein of equine, human, rabbit, porcine, and feline IgG but not of murine, rat, goat, sheep, cow or chicken IgG (Meehan et al., 2001). By interacting with the Fc interdomain region of IgG4 and IgG7, FgBP disrupts complement deposition and antibody-mediated activation of the classical complement pathway, leading to increased survival in equine whole blood, representing a host-specific immune evasion strategy of *S. equi* subsp. *equi* (Meehan et al., 2001; Lewis et al., 2008).

## Models to Study Niche- and Host-Specialized Cell Surface Proteins

The examples provided here include structural, biochemical, and molecular evidence for niche- and host-specialized interactions. However, it is often challenging to validate the importance of these host-pathogen interactions in appropriate infection models, especially when the bacteria exhibit a human tropism (Douam et al., 2015). In some cases, an effective model may not exist and the ethical and/or cost implications of performing experimental infections of large animals is prohibitive. Importantly, small animal models may not have ligands or receptors in common with the natural host and would therefore not support the function of the bacterial surface protein in question. Using knockdown and heterologous expression systems in cell lines *in vitro* can provide useful information relating to specific host–pathogen interactions but they cannot address the relevance of the interaction in a complex infection setting *in vivo*. The development of three-dimensional (3D) organoid systems derived from stem cells for infection biology is a highly promising nascent research area that will provide more complex multicellular systems for investigating host–pathogen interactions *in vitro* (Iakobachvili and Peters, 2017). One such system has been developed from murine mammary gland tissue to create a 3D mammary organoid that can be manipulated to induce lactation or involution (Sumbal et al., 2020). A similar system could be used to generate a bovine mammary organoid allowing the *in vitro* examination of the lactoferrin-dependent internalization of *S. uberis*.

**FIGURE 2:**
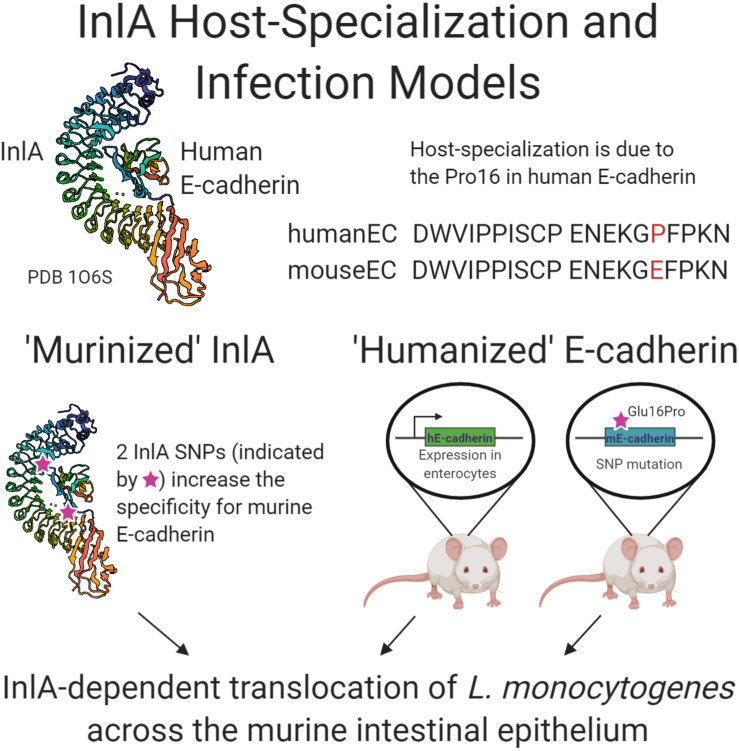
Schematic summary of research examining the interaction of internalin A of *L. monocytogenes* with human E-cadherin. Crystal structure generation of InlA in complex with human E-cadherin, PDB 1O6S (Schubert et al., 2002), allowed the “murinization” of InlA with enhanced binding to murine E-cadherin. Two transgenic murine models were developed that express the human E-cadherin protein either solely in the intestine or throughout the mouse. All three approaches demonstrated the role of InlA in crossing the intestinal epithelial barrier during *L. monocytogenes* experimental infection.

Another possibility is to construct transgenic mice that express receptors supporting the function of surface proteins. In addition to the examples already mentioned here for *S. aureus* IsdB and *S. pyogenes* M protein (Lövkvist et al., 2008; Matsui et al., 2009; Pishchany et al., 2010), this approach was carried out to examine the role of *L. monocytogenes* InlA during colonization of the gut (Figure 2). Mice were genetically manipulated to either express human E-cadherin from a specialized promoter in enterocytes or a “humanized” Glu16Pro version of the murine E-cadherin in all E-cadherin-expressing cells (Lecuit et al., 2001; Lecuit and Cossart, 2002; Disson et al., 2008, 2009). Both systems demonstrated the requirement of InlA for intestinal crossing of *L. monocytogenes* with the chimeric mice also demonstrating that both InlA and internalin B are required for placental invasion during murine pregnancy (Lecuit et al., 2001; Disson et al., 2008). In the reverse scenario, InlA on the *L. monocytogenes* cell surface has been “murinized” through two single amino acid substitutions, which enhanced binding to murine E-cadherin *in vitro* and led to infection of the murine intestinal epithelium *in vivo* (Wollert et al., 2007). This subsequently led to higher bacterial burden in multiple organs including the liver and spleen, demonstrating the importance of this InlA-dependent route of bacterial dissemination (Wollert et al., 2007). Further utilization of this “murinized” *L. monocytogenes* strain demonstrated that the C57BL/6J mouse cell line has inherent resistance to oral challenge by *Listeria* with more susceptible murine cell lines exhibiting faster dissemination of the mutated strain as well as increased cytokine production (Bergmann et al., 2013). Advances in genome editing tools, including CRISPR-Cas systems, have made transgenesis of rodents a relatively facile approach, allowing the development of new transgenic models that can facilitate investigations into the importance of host-specialized bacterial interactions *in vivo* (Qin et al., 2016). For example, recent studies have employed CRISPR-Cas edited mice expressing the human integrin component CD11b to examine the function of *S. aureus* toxin LukAB *in vivo* (Boguslawski et al., 2020).

## Conclusion

Surface proteins of Gram-positive bacteria are essential for an array of different host-pathogen interactions. Accordingly, bacterial adaptation to a new host-species or anatomical niche often requires the acquisition of new proteins or diversification of existing proteins to enable binding to polymorphic ligands or receptors. As highlighted by the examples provided in the current review, important advances have been made regarding our understanding of the molecular basis for such interactions *in vitro*. In addition, new approaches for transgenesis and mutagenesis offer great promise for the development of new organoid or whole animal models of infection that allow the dissection of the role in pathogenesis of niche and/or host-specialized surface proteins, many of which have multiple binding tropisms involving several different receptor ligands. Development of appropriate experimental models will not only allow the validation of surface proteins that play important roles *in vivo* but also improve on the current model systems used for the evaluation of novel therapeutic and vaccine candidates. The identification of alternative approaches for control of infection is of particular importance for bacteria that exhibit high levels of antibiotic resistance and are becoming increasingly difficult to treat in a clinical setting.

## Author Contributions

All authors wrote and edited the manuscript.

## Conflict of Interest

The authors declare that the research was conducted in the absence of any commercial or financial relationships that could be construed as a potential conflict of interest.
